# Perfluorocarbon-Loaded Poly(lactide-*co*-glycolide) Nanoparticles from Core to Crust: Multifaceted Impact of Surfactant on Particle Ultrastructure, Stiffness, and Cell Uptake

**DOI:** 10.1021/acsapm.4c03360

**Published:** 2025-03-03

**Authors:** Naiara Larreina Vicente, Mangala Srinivas, Oya Tagit

**Affiliations:** † Cell Biology and Immunology (CBI), Wageningen University, De Elst 1, Wageningen 6708 WD, Netherlands; ‡ Group of Biointerfaces, Institute for Chemistry and Bioanalytics, FHNW University of Applied Sciences and Arts Northwestern Switzerland, Hofackerstrasse 30, Muttenz 4132, Switzerland

**Keywords:** nanoparticles, AFM, elastic modulus, PLGA, ultrastructure, cell uptake, protein corona

## Abstract

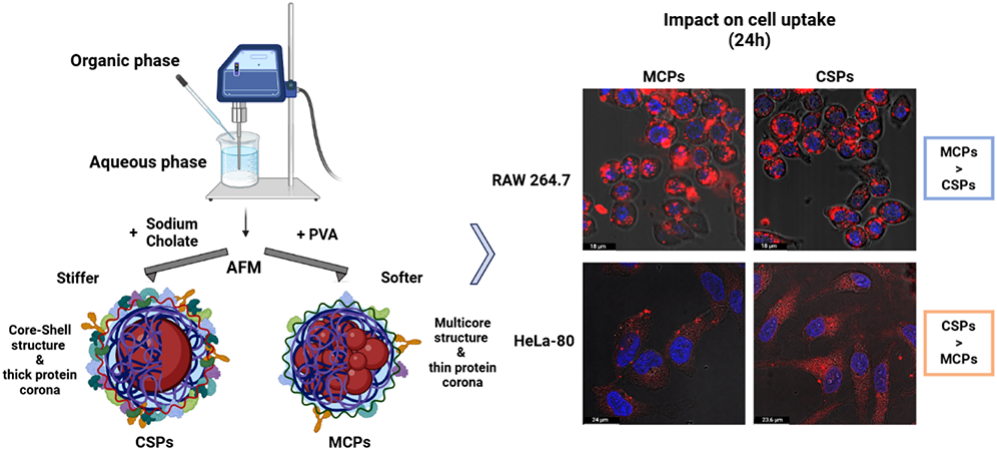

Poly(lactide-*co*-glycolide) nanoparticles (PLGA NPs) loaded with Perfluoro-15-crown-5-ether (PFCE) have been developed for imaging applications. A slight modification of the formulation led to the formation of two distinct particle ultrastructures: multicore particles (MCPs) and core–shell particles (CSPs), where poly(vinyl alcohol) (PVA), a nonionic surfactant, and sodium cholate (NaCh), an anionic surfactant, were used, respectively. Despite their similar composition and colloidal characteristics, these particles have previously demonstrated significant differences in their *in vivo* distribution and clearance. We hypothesize that these differences are collectively driven by variations in their structural, chemical, and mechanical properties, which are investigated in this study. Nanomechanical characterizations of MCPs and CSPs by atomic force microscopy (AFM) revealed elastic modulus values of 54 and 270 MPa in water, respectively, indicating a better permeability and deformability of the multicore ultrastructure. The impact of the surfactant on the NP surface chemistry was evidenced by their protein corona, which was significantly greater in the CSPs. Additionally, an important amount of residual NaCh was found on the surface of CSPs, which formed strong interactions with bovine serum albumin (BSA), accounting for the difference in protein coronas and surface chemistry. Surprisingly, *in vitro* cell uptake studies showed a higher uptake of MCPs by RAW macrophages but a preference for CSPs by HeLa cells. We conclude that for this specific formulation and in this stiffness range, mechanical differences have a stronger impact in HeLa cells, while surface properties and chemical recognition play a more important role in uptake by macrophages. Overall, the extent to which a physical factor impacts cell uptake is highly dependent on the specific uptake mechanism. With this study, we provide an integrated perspective on the role of different surfactants in the particle formation process, their impact on particle ultrastructure, mechanical properties, and surface chemistry, and the overall effect on cell uptake *in vitro*.

## 1 Introduction

With the surge of nanotechnology over the past few years, developing precision-engineered nanomedicines has become a prevalent strategy in biomedical research. Nanoparticles (NPs) represent a significant portion of these cutting-edge strategies, as they provide a chemically adaptable platform for encapsulating drugs or imaging agents, enhancing biodistribution, safety, and efficacy.^1−4^ A notable example is perfluorocarbon (PFC)-encapsulating NPs, a promising tool for noninvasive, quantitative *in vivo* imaging through ^19^F magnetic resonance imaging (MRI).^5,6^ To enable applications in diagnostic imaging, cell tracking, or targeted drug delivery,^7−9^ particle features such as size, shape, surface chemistry, and topography become crucial, as they collectively dictate the *in vivo* “fate” of the particles.^10,11^ However, these particle features are often challenging to control in certain formulations. PFCs are synthetic fluorinated organic compounds constituted exclusively of carbon and fluorine atoms. Although the high number of fluorine atoms provides optimal properties for ^19^F MRI, it also results in bulky, inert molecules of unique, simultaneously hydrophobic and lipophobic nature that form a separate phase in solution. This makes the stabilization of PFCs highly challenging and difficult to fine-tune, hindering a safe and effective translation into the clinic.^5^ Furthermore, certain PFC formulations have demonstrated significant organ accumulation rates, compromising *in vivo* biocompatibility and raising safety concerns.^12^

**Figure 1:**
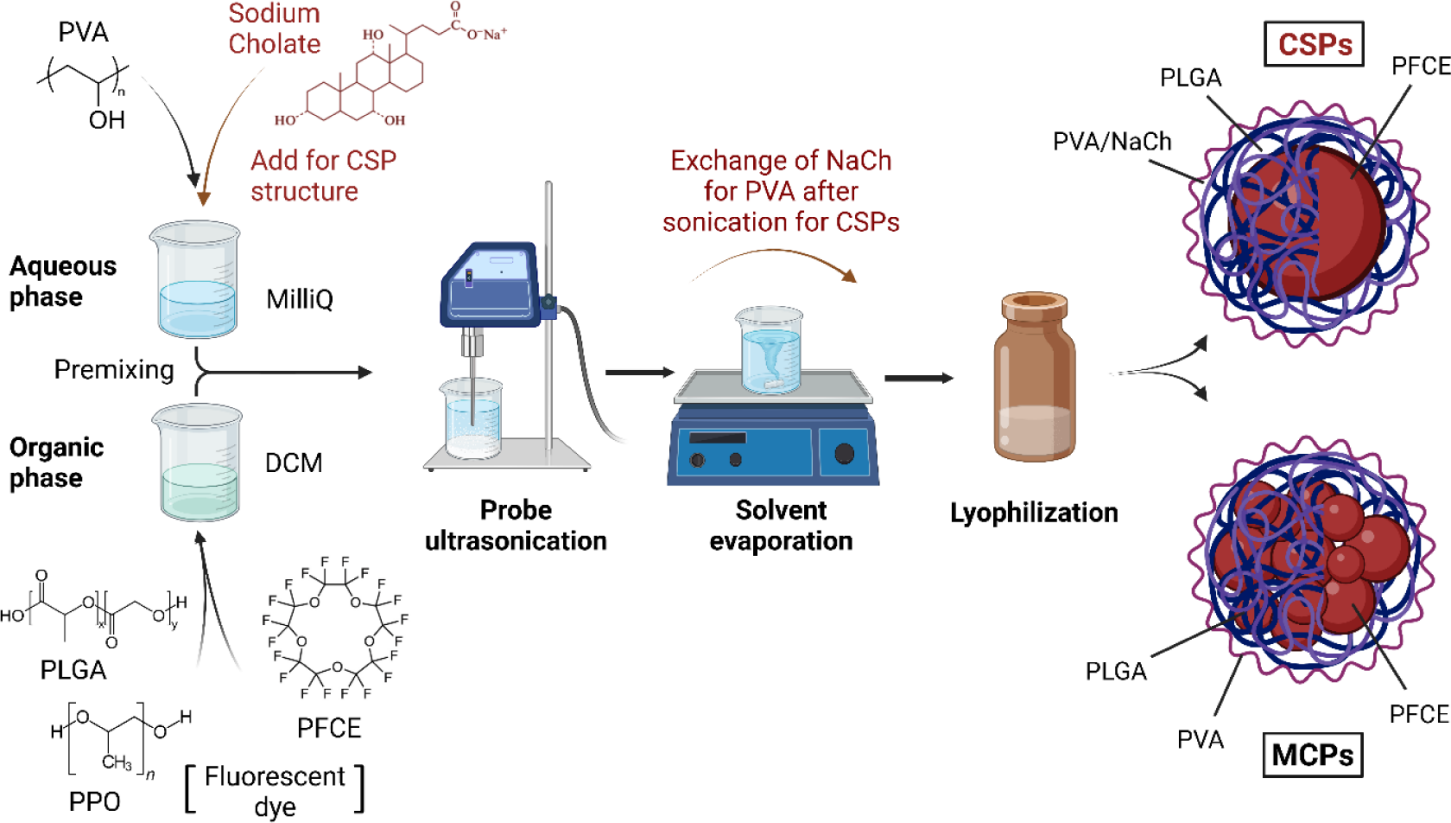
Synthetic process and structure of MCPs and CSPs, with the main formulation differences highlighted in red. For core–shell NP formation, NaCh is added as a surfactant for the sonication stage, and it is chemically exchanged by PVA during the solvent evaporation stage. For multicore NP formation, PVA remains during the whole process.

Our group has successfully developed perfluoro-15-crown-5 ether (PFCE)-loaded biocompatible, stable, and fast-clearing polymeric nanoparticles of ∼200 nm in diameter as imaging agents for *in vivo*^19^F MRI.^13−15^ The long-term stability is achieved by encapsulating PFCE in a matrix of poly(lactide-*co*-glycolide) (PLGA), using poly(vinyl alcohol) (PVA) and/or sodium cholate (NaCh) as surfactants. However, the choice of surfactant results in significant differences in particle ultrastructure (Figure 1). When PVA is used throughout the particle formation process, particles with a multicore ultrastructure (multicore particles, MCPs) are obtained.^14^ These particles exhibit a higher degree of fluorine–water interaction, revealing a higher hydration profile. In contrast, employing NaCh during the sonication step, which is later exchanged by PVA, yields particles with a core–shell structure (core–shell particles, CSPs) and reduced interaction with water.^14^ Interestingly, our previous *in vivo* work with these particles demonstrated that MCPs are cleared 15 times faster than CSPs, as observed through monitoring the ^19^F signal at accumulation sites.^11^ Considering the challenges associated with prolonged PFC retention times in clinical use, this finding highlighted the significant impact of formulation and structural differences on *in vivo* clearance rates, emphasizing the need to investigate the underlying mechanisms further.

**Figure 2:**
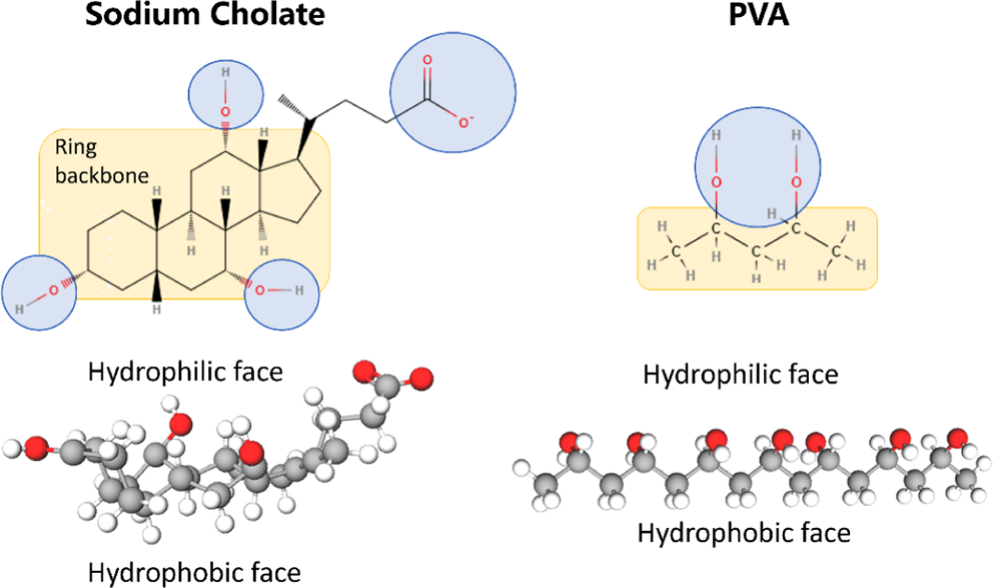
2D and 3D molecular structures and hydrophilic profiles of NaCh and PVA molecules. 3D molecules are rendered with hydrophilic moieties facing upward.

**Figure 3:**
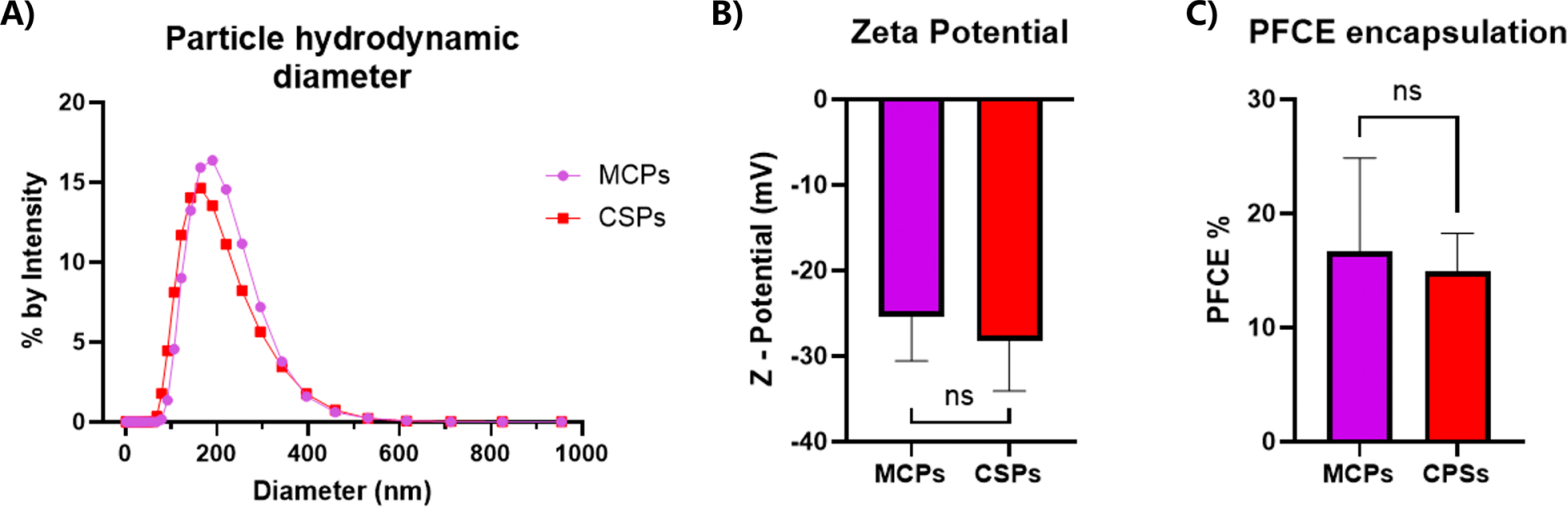
Particles were characterized in hydrodynamic diameter (A) and zeta potential (C) by dynamic light scattering (DLS), and PFCE encapsulation was measured by ^19^F NMR (B).

**Figure 4:**
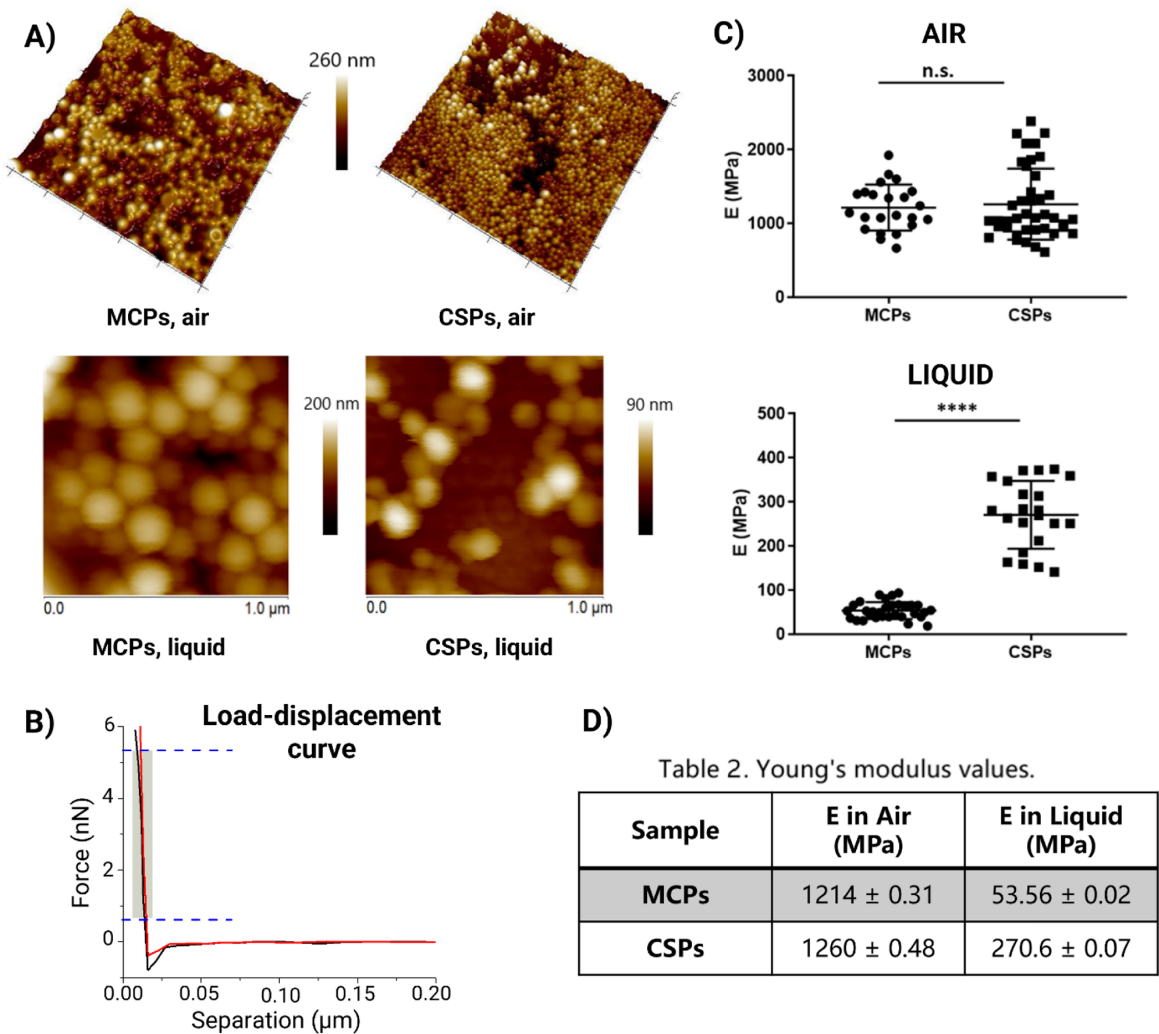
Morphological and nanomechanical characterization of MCPs and CSPs by atomic force microscopy (AFM). (A) Height images of MCPs and CSPs obtained in air and liquid. (B) Representative load–displacement curves of MCPs (black) and CSPs (red) obtained in air. The shaded part of the contact region displays the fitted portion of the curve with the linearized Sneddon’s model. (C) Distribution of Young’s modulus values of MCPs and CSPs in air and liquid, extracted from load–displacement curves obtained in the midcenter of the particles. (D) Apparent Young’s modulus of MCPs and CSPs in air and liquid, averaged from curve fit values satisfying *R*^2^ > 0.99. Similar elastic modulus values of MCPs and CSPs measured in air become significantly different for hydrated particles in liquid (*p* ≤ 0.0001).

Increasing evidence points to the role of mechanical properties of biomaterials, such as particle deformability and stiffness, in regulating particle–cell interactions, blood circulation times, and targeting efficiency.^16−19^ Studies suggest that stiffer NPs are more prone to membrane wrapping due to the higher efficiency of the engulfment process when ligand-mediated uptake occurs.^20,21^ Conversely, softer particles have been shown to exhibit faster in-cell processing following uptake,^22^ which could potentially reduce the organ accumulation time of PFCs. Guo et al. showed that soft NPs (Young’s modulus <1.6 MPa) exhibited greater uptake and tumor accumulation compared to their stiffer counterparts (Young’s modulus >13.8 MPa), due to their ability to deform and diffuse through cancerous tissue.^23^ Conversely, Hui et al. reported that softer, more deformable particles hindered phagocytosis by macrophages and cancer cells.^19^ A recent study on PFC NPs also highlighted the superior *in vivo* performance of soft polymeric NPs, which displayed gradual clearance unlike a stiffer silica-based formulation.^24^ These findings suggest that mechanical properties play a crucial role in various stages *in vivo*, which can benefit or hinder interactions with different cellular targets according to the therapeutic goals. For instance, prolonged circulation in the bloodstream is oftentimes desirable for noninvasive cardiovascular imaging or enhancing nanoparticle biodistribution to target areas in precision medicine therapies. On the contrary, uptake by immune cells might be of interest for immunotherapies or monitoring inflammation stages of a certain disease.

In addition to mechanical properties, surface chemistry and the protein corona also play key roles in nanoparticle delivery, uptake, and *in vivo* clearance. The protein corona, a dynamic multilayer of proteins that accumulate on the surface of nanoscale biomaterials, forms through strong (hard) and weak (soft corona) interactions. At the cellular level, the protein corona composition can regulate the mechanisms and efficiency of cell uptake and intracellular processing. Evidence suggests that the composition and presence of specific proteins govern the uptake by particular cell types.^25−29^ Depending on its nature, the surfactant can facilitate interactions with specific proteins via electrostatic or hydrophobic interactions, directly impacting the protein corona composition. In addition to particle surface chemistry,^30−32^ the particle mechanical properties have been suggested to impact the composition of protein corona^33^ with stiffer nanocarriers adsorbing more opsonins.^34^

The examples discussed above point out the intricate influence of particle mechanical and physicochemical surface properties on their cell uptake and *in vivo* clearance patterns. While it is known that the surfactant of choice might trigger or avoid certain clearance pathways,^32^ it is yet to be explored how it can affect particle ultrastructure and the physicochemical properties derived from it. In this study, we demonstrate the multifaceted impact of the surfactant type on the structural, mechanical, and surface properties of PFCE-loaded PLGA nanoparticles. A slight modification of the formulation in terms of surfactant type led to different distribution profiles of the PFCE phase through the polymer matrix with direct implications on the nanoparticle internal structure. While the nonionic surfactant PVA facilitated the formation of multicore particles with the PFCE phase distributed as multiple small domains through the nanoparticle (multicore particles, MCPs), the anionic surfactant NaCh packed the PFCE phase as a single domain within the core of the nanoparticles (core–shell particles, CSPs). This difference in the nanoparticle ultrastructure further impacted the hydration profile of the MCPs and CSPs, giving rise to significantly different nanomechanical properties, as demonstrated by atomic force microscopy measurements. Detailed investigations elucidated the influence of surfactant type on the protein corona formation and the resulting differences in uptake profiles by phagocytic and nonphagocytic cells. We conclude that for this specific formulation and in this stiffness range, mechanical differences have a stronger impact on the uptake by HeLa cells, while surface properties and chemical recognition play a more important role in the uptake by macrophages. Overall, our study highlights the importance of optimizing surfactant selection for designing nanoparticles with tailored properties for specific applications, such as drug delivery, imaging, or diagnostics, where uptake efficiency by the biological targets is paramount.

## 2 Experimental Section

### 2.1 Materials and Reagents

PLGA (Resomer RG 502 H) was purchased from Evonik Röhm GmbH, Germany. Perfluoro-15-crown-5-ether (PFCE, 99%, Prod. ID# 080010) was acquired from Exfluor Research Corporation, USA. Dimethyl sulfoxide (DMSO, ≥99.9% USP) was supplied by J.T. Baker, USA; dichloromethane (DCM, ≥99.5% ACS) was acquired from VWR Chemicals BDH, USA; and boric acid (Cat. No. 0588, ACS) was purchased from VWR Life Science, USA. Poly(vinyl alcohol) (PVA, Mw 9 000–10 000, 80% hydrolyzed, Cat. No. 360627), bovine serum albumin (BSA, ≥98%, Cat. No. A9647), Cell Counting Kit-8 (Cat. No. 96992), paraformaldehyde (PFA, powder 95%, Cat. No. 158127), sodium hydroxide (Cat. No. 106498), deuterium oxide (Cat. No. 151882), and trifluoroacetic acid (TFA, Cat. No. T6508) were obtained from Sigma-Aldrich, Merck KGaA, Germany. Iodine–potassium iodide solution to Lugol (Art. No. N052.1) was obtained from Carl Roth GmbH + Co. KG, Karlsruhe, Germany. Atto-Oxa12 (AD Oxa12–25) was purchased from ATTO-TEC GmbH, Germany. Zombie Aqua Fixable Viability Kit (Cat. No. 423102) was obtained from BioLegend, San Diego, USA, and EasyProbe Hoechst 33342 Live Cell Stain (Cat. No. FP027) was acquired from ABP Biosciences, Beltsville, USA. Dulbecco’s modified Eagle medium (DMEM, high glucose −4.5 g/L, Cat. No. 11965092) was purchased from Gibco, Thermo Fisher Scientific, USA. Pierce Coomassie Brilliant Blue (G-250; Cat. No. 20279), hydrochloric acid (37% solution in water, Cat. No. 10794821), and sodium cholate hydrate (99%, Cat. No. A17074.18) were supplied by Thermo Fisher Scientific Inc., USA. Glass High Precision Cell cuvettes (Art. No. 100–10–20) were acquired from Hellma Analytics, Müllheim, Germany.

### 2.2 Synthesis of Multicore NPs

Nanoparticles were made as described previously.^14^ Briefly, PLGA (100 mg, Resomer RG 502 H, lactide:glycolide molar ratio 48:52 to 52:48; Evonik Industries, Germany) was dissolved in dichloromethane (DCM) (3 mL) and mixed rapidly with PFCE (900 mL, Exfluor Inc., TX, USA). This mixture was added rapidly to an aqueous solution of poly(vinyl alcohol) (25.5 g, 1.96 wt %) and emulsified for 3 min under sonication at 40% amplitude using a digital sonicator from Branson Ultrasonics (Connecticut, USA). The solvent was evaporated overnight at 4 °C under stirring, and nanoparticles were collected by centrifugation 16 000 g for 35 min, washed thrice with distilled water, and lyophilized.

### 2.3 Synthesis of Core–Shell NPs

To synthesize core–shell nanoparticles, PLGA (100 mg, Resomer 502H) was dissolved in DCM (3 mL) and mixed with PFCE (900 mL) by pipetting it up and down with a glass pipette. The resulting primary emulsion was added to a solution of NaCh (25 g, 1.5 wt % solution in water) and sonicated on ice for 3 min at an amplitude of 40% (Branson Digital Sonifier S250). After sonication, DCM was evaporated overnight under stirring at room temperature. To exchange the surfactant, a PVA solution (10 g of 1.96 wt % solution) was added to the suspension and the mixture was stirred at 4 °C for 3 days. The particles were washed thrice with distilled water at 16 000 g for 35 min. After washing, particles were resuspended in water (4 mL), frozen with liquid N_2_, and freeze-dried.

### 2.4 Colloidal Characterization

#### 2.4.1 Dynamic Light Scattering (DLS)

The hydrodynamic diameter and polydispersity index (PDI) of the particles were measured by DLS on a Malvern Zetasizer ZS Nano instrument (Malvern, UK). Samples were measured at a concentration of 0.01 mg/mL in a glass cuvette using the backscattering mode.

### 2.5 Nanoparticle Composition

#### 2.5.1 PFCE Content: ^19^F Nuclear Magnetic Resonance Imaging (NMR)

The PFCE content of MCPs and CSPs was measured by ^19^F NMR on a 9.4 T Bruker Avance III 400 MHz instrument equipped with a BBFO probe. MCPs or CSPs were dissolved in D_2_O (Sigma-Aldrich, Germany), and a 1% TFA (trifluoroacetic acid) (Sigma-Aldrich, Germany) internal reference was added. The mixture was measured at −85 ppm with a sweep of 40 ppm and 8 averages with an interscan delay of 25 s. NMR spectra were processed using MestReNova (version 10.0.2, Mestrelab Research).

#### 2.5.2 PVA Content

To measure the PVA content of both particle types, different batches of various PFCE % and sizes were chosen to diversify the samples and minimize statistical biases. The amount of residual PVA was determined by a colorimetric method based on the formation of a colored complex between two adjacent hydroxyl groups of PVA and an iodine molecule. Briefly, 500 μL of the sample suspensions were treated with 200 μL of 0.5 M sodium hydroxide solution for 15 min in a bath sonicator (BANDELIN electronic GmbH & Co. KG, Berlin, Germany) at 60 °C. Afterwards, samples were neutralized with 90 μL of 1 M hydrochloric acid. To each sample, 300 μL of a 0.65 M solution of boric acid and 50 μL of Lugol’s iodine solution (I_2_/KI) were added. Finally, after 15 min of incubation at 700 rpm, absorbance was measured at 689 nm using an Infinite M200 PRO (Tecan, Männedorf, Switzerland) plate reader. A standard curve of known PVA concentrations was prepared in every replicate (*n* ≥ 3) and was used for calculating the amount of PVA present in the unknown samples.

#### 2.5.3 Detection of Sodium Cholate by NMR

The presence of NaCh in CSPs was detected through ^1^H NMR on a 700 MHz standard-bore NMR spectrometer (Bruker Biospin), equipped with a Broadband Inverse (BBI) probe at a set temperature of 298 K. To do so, 5 mg of different CS and MC particle batches (*n* ≥ 3) were solubilized in 600 μL of DMSO-*d*_6_ and bath sonicated for 30 s. Control samples of PLGA, PVA, and NaCh were prepared at comparable concentrations to identify the peaks in the composite nanosystems. The obtained spectra were processed and aligned with MestReNova (version 10.0.2, Mestrelab Research).

### 2.6 Morphological and Nanomechanical Characterization

Atomic force microscopy (AFM) images of MCPs and CSPs were obtained by using a Catalyst BioScope (Bruker) atomic force microscope coupled to a confocal microscope (TCS SP5II, Leica). A 100 μL of 1 mg/mL particle suspension was dried on clean glass substrates, and particles were imaged in peak-force tapping mode. Silicon nitride cantilevers with nominal spring constants of 0.4 or 0.7 N/m (Bruker) were used for the imaging of MCPs and CSPs in air and fluid, respectively. The scan rate was set at 1 Hz. 256 lines with 256 points per line were recorded during image acquisition. AFM images were analyzed by using NanoScope analysis software (Bruker).

Quantitative nanomechanical mapping (QNM) was used for the mechanical characterization of MCPs and CSPs. This mode has the advantage of simultaneously providing high-resolution images and force measurements of the particles. Silicon nitride cantilevers with a nominal spring constant of 0.7 N/m (Bruker) and a nominal tip radius of 20 nm were used without any tip modification. The system was calibrated in air and water before each experiment by measuring the deflection sensitivity on a hard substrate, which enabled the subsequent determination of the cantilever spring constant using the thermal tune method.^35^ 128 × 128 force curves (a total of 16 384 curves) per scanned area were recorded, and those obtained in the middle of the particles were extracted from force-volume images and exported for further analysis using NanoScope analysis software (Bruker). After baseline correction, approach curves were analyzed to determine Young’s modulus of elasticity using Sneddon’s conical indenter model^36^ for which Poisson’s ratio was set as 0.5 and the half angle of the indenter as 18°. The contact point-independent linearized Sneddon equation was used for fitting the approach curves.^37^ The model was fit on the approach curve by setting the lower and upper fit boundaries to approximately 10% and 70% of the maximum exerted force, respectively. Fit values satisfying *R*^2^ > 0.99 were averaged to determine the apparent Young’s modulus of elasticity of MCPs and CSPs in air and liquid.

### 2.7 Surface Characterization

#### 2.7.1 Protein Corona

**Figure 5:**
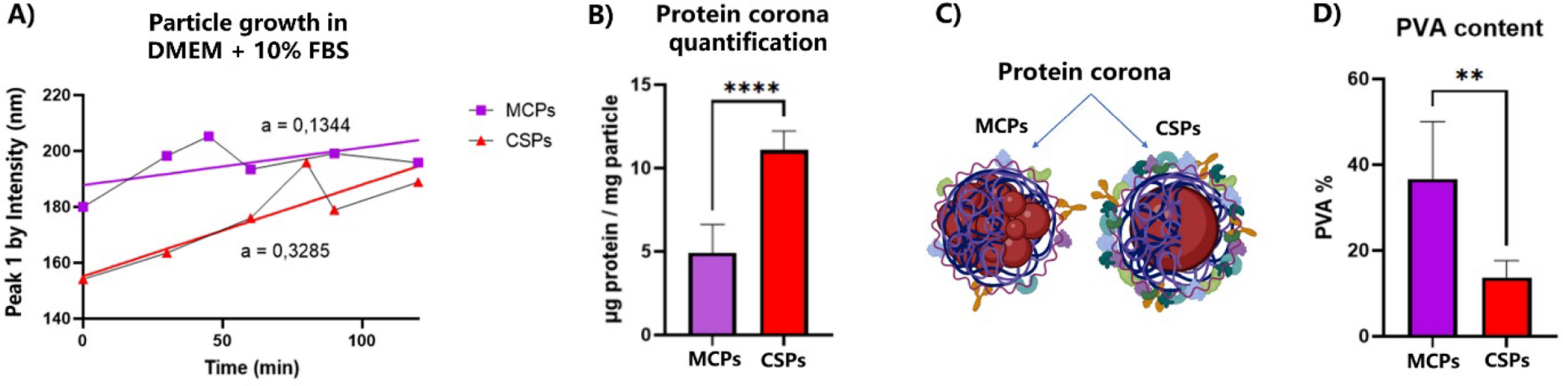
Protein corona formation. (A) Changes in hydrodynamic diameter (Peak 1 by intensity) in cell culture medium measured by DLS (*a* = slope of the trend line). (B) Protein adsorption (hard corona) in MCPs and CSPs quantified by the Bradford assay; the protein corona is significantly different in both particles (*p* ≤ 0.0001). (C) Representation of particle structure and protein corona in biological environments. (D) Average PVA % in MCPs and CSPs, quantified by a colorimetric assay, is significantly different, revealing over double the amount in MCPs (*p* ≤ 0.01).

Two methods were used to quantify the protein adsorption rate and the amount of proteins bonded as part of their hard corona. First, we monitored particle growth through dynamic light scattering with a Malvern Zetasizer ZS Nano instrument (Malvern, UK). Different NP samples were suspended at a concentration of 1 mg/mL in 10% FBS-supplemented DMEM and were incubated at a 37 °C bath for 2 h. One mL of sample was transferred into a glass cuvette for measurement at 15–30 min intervals. Measurements were performed at the same temperature, and Peak 1 by intensity is plotted in the graph (Figure 5A) to monitor the particle population and avoid the signal from free protein agglomerations. Control samples of medium and NPs in ultrapure water were used as a reference to identify the peaks in the mixed samples.

To determine the amount of protein bound to the particles, a slightly modified version of the Bradford assay protocol described in the study^38^ was followed. Briefly, the particles were incubated in MQ + 20% FBS medium at a 4 mg/mL concentration for 1 h at 37 °C and were subsequently washed through consecutive centrifugation steps. Particle pellets were degraded by dissolution in 250 μL NaOH under shaking at 1200 rpm at 60 °C for 15 min. 240 μL of Pierce Coomassie Brilliant Blue (Thermo Fisher Scientific Inc., USA) was added to 50 μL of sample solution and incubated at RT in a 96-well plate, followed by absorbance measurement at 595 nm with a CLARIOstar Plus spectrophotometer (BMG LABTECH, Germany). A control curve of known concentrations of BSA was used to quantify the protein amount in test samples. The recorded values were normalized per mg of particle in each sample, and *n* > 3 batches were used for statistical analysis (Student’s *t* test) (Figure 5B).

#### 2.7.2 Particle–Protein Interaction: Fluorescence Spectroscopy

To detect NaCh on the surface of CSPs and monitor the interaction with BSA, we used fluorescence spectroscopy following the intrinsic fluorescence of Trp residues of BSA. Based on the observed effect in the fluorescence of BSA when interacting with NaCh, shown in other studies,^39^ we first aimed to find the perfect BSA/NaCh ratio to monitor the interaction. Several 5 μL BSA solutions were prepared in MQ water with growing concentrations of NaCh (0–2.5 mM), with the pertinent controls. Spectral fluorescence curves were acquired with an Infinite 200 PRO Microplate reader (Tecan Trading AG, Switzerland) at 270 nm excitation and 280–350 nm emission. The same protocol was followed with PVA to assess the effects of both surfactants. Equal solutions of NaCh and PVA in MQ water were prepared to subtract the background fluorescence from the BSA-surfactant complex samples.

To detect the presence of NaCh on the surface of CSPs, BSA solutions at 5 μL were prepared and growing concentrations of CSPs and MCPs were added (2–4 mg/mL). Control particle solutions were prepared in ultrapure water at the same concentrations to extract the baseline fluorescence of the particles.

### 2.8 Cell Uptake Studies

RAW 264.7 and HeLa-80 cell lines were used for the cell uptake studies *in vitro*. Both cell lines were cultured in a humidified chamber at 37 °C and 5% CO_2_ for 2 weeks before the experiments for preconditioning, in Dulbecco’s modified Eagle medium (DMEM) supplemented with 10% FBS, 100 U of penicillin, and 100 μg/mL streptomycin. No FBS was added to the medium for the serum-free experiments. For the particle uptake studies, different batches with the same fluorescent dye (AttoOxa12) and similar size, zeta potential, and PFCE encapsulation were preselected. From these batches, the fluorescence intensity of equal-concentration solutions was measured with a plate reader, and two MCP and CSP batches with highly similar fluorescence intensity were selected for the experiments (Figure S1).

For the time point experiments, the cells were plated the day before in 12- or 24-well culture plates, and a constant particle concentration of 1 mg/million cells was added at time zero, with three replicates of each condition. At different measuring time points, the particles were washed 3 times with PBS, and samples were prepared for the applicable analysis method. When time points were close (0–90 min for RAW macrophages), the NPs were added at different time points and fixed at the same end point.

#### 2.8.1 Flow Cytometry

Cell uptake was quantified by flow cytometry with a Cytek Aurora instrument (Cytek Biosciences, Fremont, CA, USA). For quantification, the cells were gathered, fixed, and stained with the viability marker Zombie Aqua. The samples were transferred to a 96-well plate for FC automated analysis with the pertinent controls. For the data analysis, the cell populations were manually gated using the control samples as a reference and following a standardized workflow: FSC/SSC > Live/Dead > Positive/Negative.

Either the average fluorescence intensity or the positive population % was used as the study parameter for the comparative analysis. The uptake curves over time are plotted using the average normalized intensity values.

For the serum-free experiments, the median fluorescence intensity of the live cell population in both serum-supplemented and serum-free conditions was used to calculate the uptake ratio following the equation:
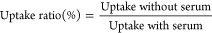


The uptake ratio was calculated in every replicate test for each particle type and cell line (*n* ≤ 3), and one-sample *t* tests were performed for statistical analysis.

#### 2.8.2 Confocal Imaging

For the confocal imaging of the labeled cells, in-house engineered 8-well LabTek Chamber Slides (Nunc, Langenselbold, Germany) with a thin bottom were used. The above-mentioned protocol was followed for the particle uptake assay, and Hoechst EasyProbe (ABP Biosciences, Beltsville, USA) was applied to stain the nuclei 15 min before washing and fixing the cells with paraformaldehyde (PFA) 4% for 20 min. A Leica TCS SP8 X White Light Laser inverted confocal microscope (Leica Microsystems B.V., Amsterdam, Netherlands) was used for imaging, with an HCX PL APO 63×/1.4 NA oil-immersion objective (12-bit resolution, 1024 × 1024 pixels, 1400 Hz speed) and LasX software for image processing. The imaging settings were kept constant between samples, and the same postprocessing method was applied for analysis.

#### 2.8.3 Viability Studies

WST-8 colorimetric assay was performed with the Cell Counting Kit-8 reagent (Merck, Germany) to assess the effects on viability of incubating the cells in a serum-free medium. 10 000 (HeLa) or 25 000 (RAW) cells were plated in a 96-well plate a day before the experiment. The samples were incubated at different time points under serum-supplemented and serum-free conditions. For the analysis, 10 μL of WST-8 reagent was added to 100 μL of medium with or without serum and incubated for 1 h. Consecutively, the media were transferred to black well plates for spectrophotometric analysis in a CLARIOstar microplate reader (BMG LABTECH, Germany), where the absorbance at 450 nm was quantified as the reference value of the viability. For the analysis, the values were normalized to the control conditions, and an ordinary one-way ANOVA with multiple comparisons was conducted to assess the results.

#### 2.8.4 Statistics and Graphs

Each experiment was repeated at least thrice with 3–4 technical replicates. All graphs and statistical analyses were performed using GraphPad. The test type and *p*-values are shown in the caption of each figure. The graphs show the mean and standard deviation of the normalized or transformed raw values.

## 3 Results and Discussion

### 3.1 Particle Preparation and Characterization

Numerous batches of multicore and core–shell nanoparticles were prepared as described in the “Experimental Section”. Briefly, the particle synthesis process employs probe sonication as the emulsifying method, which provides sufficient energy to entrap bulky PFCE molecules in the PLGA matrix. Due to the highly hydrophobic and lipophobic nature of PFCs, high-energy emulsification methods and strong stabilizing agents are required for their encapsulation. As shown in Figure 1, the primary components for both particle types are very similar: PLGA (50:50 lactide:glycolide ratio) as the polymer matrix, PFCE as the imaging agent, and PVA or NaCh as surfactants.^14^ A small amount of poly(propylene oxide) (PPO) is also incorporated to enhance PFCE encapsulation efficiency, acting as an additional stabilizer during the sonication step.

In both cases, the raw materials, sonication time, and energy output were kept constant, and particle formation was achieved via the emulsion-solvent evaporation technique (SET).^40^ The technique relies on the gradual removal of the volatile organic solvent as it evaporates from the o/w emulsion, inducing particle solidification and precipitation. Since these particles may be applied *in vitro*, some batches incorporate hydrophobic fluorescent dyes in the organic phase to enable analysis by fluorescence microscopy and flow cytometry.

There is a key distinction that leads to the formation of these two NP structures. MCPs are produced using PVA as the stabilizer throughout the entire particle formation process, whereas CSPs are synthesized using NaCh during the sonication step, which is later replaced by PVA following solvent evaporation. For the surfactant exchange, a 1.96% (w/v) PVA solution is added to the NP suspension and maintained stirring at 4 °C for 3 days, allowing for the gradual substitution of NaCh by PVA while preserving the original nanoparticle structure.

PVA is a synthetic amphiphilic nonionic polymer. Its linear structure, composed of both hydrophilic and hydrophobic moieties, forms an adaptable interconnected network with the polymer matrix, effectively reducing the interfacial tension with the aqueous phase.^32,41,42^ Contrarily, the bile salt NaCh is an amphiphilic anionic surfactant composed of a quasi-planar rigid steroid ring backbone as a hydrophobic face and a hydrophilic face formed by hydroxyl groups and a charged carboxylate group (Figure 2). Due to this rigid yet slightly bent structure, NaCh adapts well to curved surfaces, leading to the formation of core–shell structured systems.^43−46^ While the hydrophilic nature of PVA minimizes nonspecific interactions with proteins and solutes,^47^ bile salts, particularly NaCh, can form strong interactions with proteins such as BSA.^39,48^ Thus, the differential presence of these surfactants on the particle surface might influence the interactions with proteins and cells. Additionally, properties such as viscosity, solubility, flexibility, and hydrophobic surface adhesion are tunable in PVA, as they depend on its polymerization degree and molecular weight,^49^ whereas the rigid structure of NaCh presents localized hydrophobic interactions due to the steroid backbone and its monomeric nature.

To evaluate how this variation affects the final product, multiple batches were characterized for hydrodynamic diameter (via DLS), zeta potential (via DLS), PFCE content (via ^19^F NMR; Figure S2), and fluorescence intensity, when applicable (Figure 3 and Table 1). The hydrodynamic diameter distributions, depicted in Figure 3A as the percentage of particles by intensity, show that both particle types exhibit similar size profiles, with the main population being just under 200 nm. The mean zeta average value indicates a slightly larger average for particles formulated with PVA (Table 1). A similar effect on particle size was reported by Esim et al. when using NaCh and PVA as surfactants for PLGA NPs.^43^ Regarding their zeta potential, although the average in CSPs seems to be slightly lower, no statistically significant differences were found (Figure 3B). We would expect a more negative surface charge in CSPs if significant amounts of NaCh remained on their surface, due to their anionic nature. Therefore, we hypothesize that CSPs adsorb sufficient PVA during the surfactant exchange process to achieve a zeta potential comparable to the MCPs. Additionally, in the cited study, they report instability and aggregation problems when using NaCh as the surfactant for PLGA nanoparticles,^43^ something we have also encountered in numerous CSP batches. Both structures entrap similar amounts of PFCE (Figure 3C).

**Table 1:** Colloidal Characteristics of MCPs and CSPs (*n* ≥ 6)

Sample	Diameter (nm)	PDI	z Potential (mV)	PFCE (%)
MCPs	185 ± 16	0.11 ± 0.03	–25 ± 5	17 ± 8
CSPs	168 ± 25	0.08 ± 0.04	–28 ± 5	15 ± 3

### 3.2 AFM Studies

To investigate the impact of structural differences on the mechanical properties of these NPs, atomic force microscopy (AFM) studies were conducted. First, particles deposited on glass substrates were imaged in both air and liquid environments to examine their topography and morphology (Figure 4A). AFM height images revealed a smooth, spherical morphology for both particle types, consistent with the monodisperse populations and low polydispersity indices derived from DLS analysis.

To assess particle stiffness, the Young’s modulus was calculated using the force curves (Figure 4B) acquired on top of the particle centers using quantitative nanomechanical mapping (QNM). The Young’s modulus quantifies the stress or force required to induce material deformation. Therefore, lower values indicate more elastic, deformable, or softer biomaterials. Particle stiffness was measured in the dry and hydrated states to evaluate how hydration influenced elasticity. Given the complexity and heterogeneity of these composite nanosystems, we refer to the derived elastic modulus values as the apparent Young’s modulus, as different layers and components of the particles likely exhibit different rigidity. The utilization of the linearized Sneddon model to fit the approach curves obtained on the top-middle part of the nanoparticles could reflect the local apparent elastic modulus of our samples with complex and heterogeneous structures. For a detailed description of the analysis approach, see Figure S3.

As shown in Figure 4C,D, air-dried MCPs and CSPs displayed very similar mean apparent Young’s modulus values (1240 and 1260 MPa, respectively). Statistical analysis revealed no significant differences in the dry state, which is consistent with their comparable chemical composition and highlights the fact that their components share intrinsically similar mechanical properties. In contrast, we observe a substantial difference in stiffness in the hydrated state, with values of 54 MPa for MCPs and 270 MPa for CSPs. As noted, MCPs exhibited greater interaction between fluorine and water molecules in solution,^14^ supporting the hypothesis that they present a more permeable structure and a higher hydration profile. Overall, these findings suggest that particle stiffness in solution is dictated by differences in permeability that are driven by the distribution of the PFCE phase within the polymer matrix. Of note, other factors such as the ionic strength and hydrophobicity of surfactants could also affect permeability by influencing the adsorption of water molecules, which still requires investigation.

The higher deformability exhibited by multicore particles could influence processes like cell adhesion, particle engulfment, and intracellular processing after uptake.^19,20^ More deformable particles (<1.6 MPa) would also present a superior ability to deform and diffuse through the narrow cavities of cancerous tissue, affecting their accumulation rate and overall uptake.^23^ Thus, particle stiffness and deformability could affect not only the uptake process but also passive factors such as the enhanced permeability and retention (EPR) effect.^50^ However, the absolute values of these particles’ Young’s moduli would be considered substantially high relative to the biological environment. Macrophages can recognize and respond to altered mechanical properties,^51^ sensing elements like fibrotic tissue (stiffness >20 kPa)^52^ and tumors (∼100 kPa)^53,54^ (mechanosensing), or aged erythrocytes (∼50–100 kPa)^55,56^ for phagocytosis. Therefore, materials with stiffness exceeding 100 kPa would be considered significantly rigid (see Table 1 of Lee et al.^57^), which is an order of magnitude lower than the particles under study. The mechanisms by which rigidity takes action at these high ranges might differ substantially.^58^ The existing literature on the effect of stiffness on endocytosis is based on synthetic particles with varying elastic modulus ranges, sizes, and compositions, where all parameters act in synergy.^59,60^ At these stiffness ranges, it remains unclear whether a difference between 54 and 270 MPa is relevant for cells,^51^ keeping in mind that the measured nanoscale mechanical properties of these complex and heterogeneous particles may not fully reflect the actual stiffness “sensed” by cells.

### 3.3 Particle Surface Characterization: Surfactant and Protein Corona

Since the particles exhibit slight differences in formulation in terms of the surfactant, we investigated their surface composition and *in vitro* uptake. For this purpose, we analyzed the protein corona by tracking changes in particle size over time in serum-supplemented cell culture media (10% fetal bovine serum, FBS) to monitor the adsorption rate of serum proteins. As indicated by the linear regression slope, CSPs exhibited a more pronounced size increase than did MCPs (Figure 5A). These measured changes reflect both the hard corona and the loosely bound soft corona. The hard corona was quantified following a 1 h incubation in DMEM and subsequent washing to remove the loosely bound proteins. A colorimetric assay (Bradford assay^38^) revealed that CSPs adsorbed more than twice the amount of protein compared to MCPs (Figure 5B). Multiple studies^33,34,61^ have reported the influence of nanoparticle stiffness on protein adsorption rates, which could explain the observed differences between MCPs and CSPs (Figure 5C). Nevertheless, given the differences in the surfactant composition, we further explored the surface characteristics of both particle types.

To assess the efficiency of the surfactant exchange process and investigate the surface composition, we measured the PVA content across different particle batches as described in the “Experimental Section”. The results revealed significantly higher levels of PVA in MCPs, which could be explained by the need to stabilize multiple PFCE cores throughout the PLGA matrix (Figure 5D). However, given the significant difference between the particle types, we investigated the presence of residual NaCh in CSPs, which could account for their elevated protein adsorption.^62^

**Figure 6:**
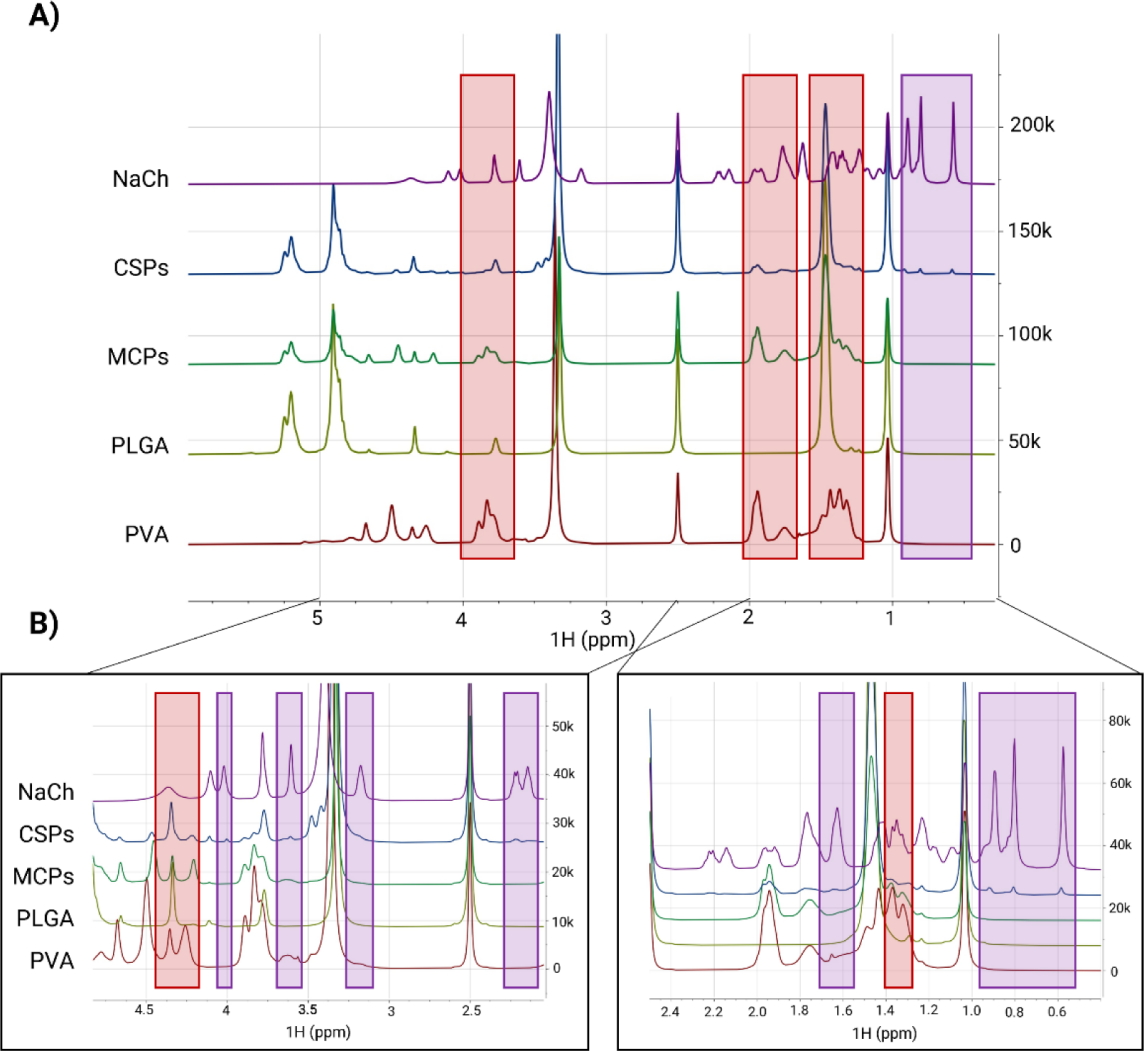
(A) ^1^H NMR spectra of NaCh, CSPs, MCPs, PLGA, and PVA, from top to bottom. Samples of the different particle components were independently analyzed in DMSO. The superimposed display of the aligned spectra shows differences in specific peaks, revealing the presence of NaCh traces in CSPs that are not visible in MCPs (blue boxes) and a significantly higher signal of PVA in MCPs, compared to CSPs (red boxes). (B) The general spectra are zoomed in on different areas for better visualization. The data were analyzed and plotted with MestReNova.

Particle composition was analyzed by ^1^H NMR (Figure 6), and the spectra of both particles were compared with their individual components. Interestingly, the ^1^H NMR spectra revealed substantial amounts of residual NaCh in CSPs, suggesting an incomplete surfactant exchange with PVA. Over three different batches of each particle type were tested, providing consistent evidence (Figure S4A,B). As a result, significant differences might be found in particle surface chemistry, even though the zeta potential values were similar. Previous studies have shown strong hydrophobic interactions between cholates and albumin, one of the main proteins of serum.^48,63,64^ On that basis, we would expect to see an interaction between CSPs and albumin in culture media if NaCh was present near the particle surface, leading to higher protein adsorption *in vitro*. To confirm this, we monitored the intrinsic fluorescence of the tryptophan (Trp) residue of BSA under different conditions. In the mentioned studies, NaCh was shown to have a stabilization effect on BSA, which was detected as a blue-shift in peak emission wavelength (λ_max_) and fluorescence quenching. This effect is produced due to the more hydrophobic environment that the NaCh molecules provide when arranged around the Trp residues.^64^ Based on this effect, we developed a method to confirm whether NaCh is present on the particle surface and interacts with serum proteins.

**Figure 7:**
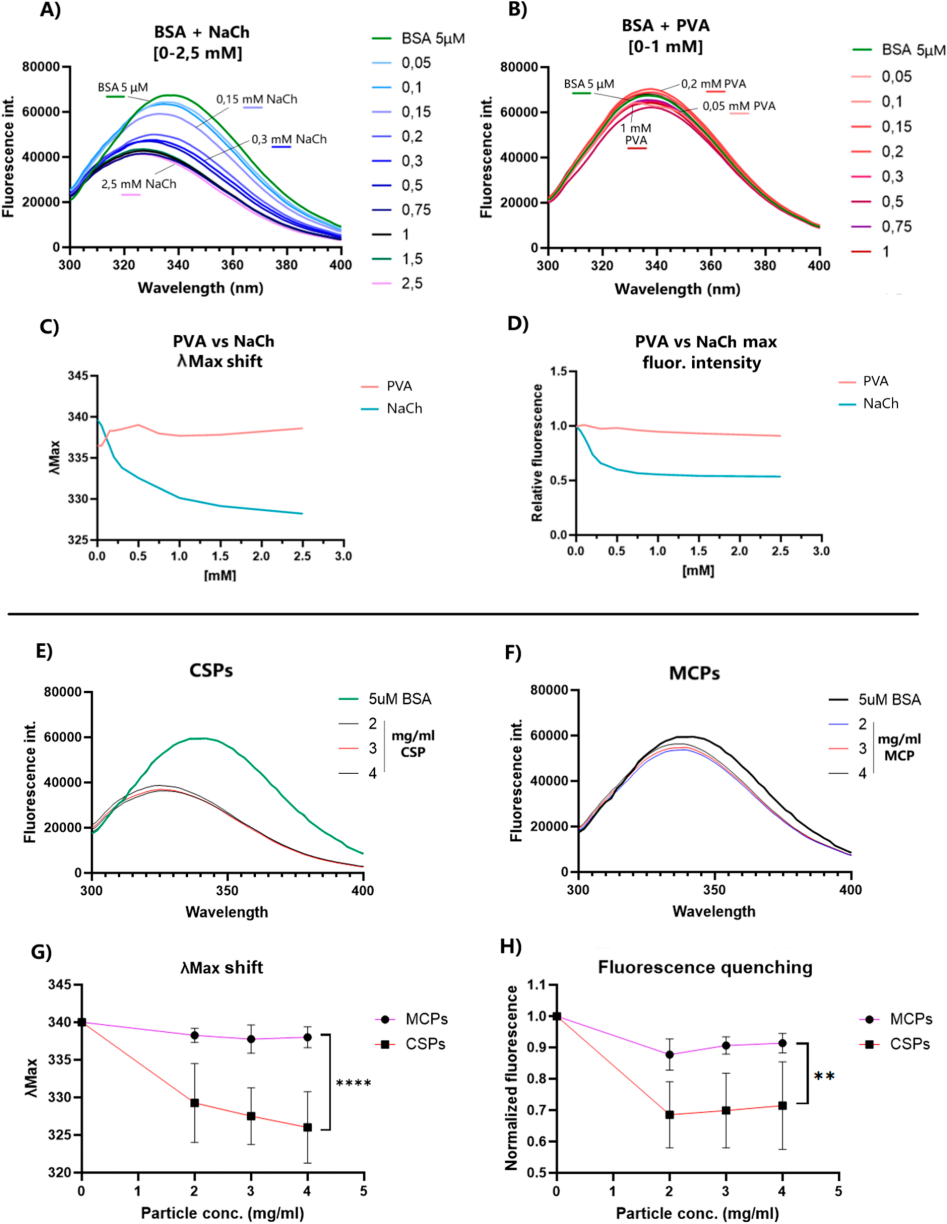
NaCh–BSA interaction studies. Spectral fluorescence emission (ex. 270 nm) of BSA 5 μM with growing concentrations of PVA (A) and NaCh (C) following the intrinsic fluorescence of the Trp residue of BSA in different chemical environments. Effect on the quenching (intensity decay) and λ_max_ (change in wavelength) of BSA–NaCh (B) and BSA–PVA complexes (D) at growing surfactant concentrations. Spectra of CSPs (E) and MCPs (G) at different concentrations (2–4 mg/mL) in addition to BSA (final concentration of 5 μM), and the observed effects in peak shift (F) and quenching (H). The fluorescence quenching and blue-shift produced by the NaCh–BSA interaction are also detected with CSPs–BSA, with statistically significant differences compared to the effect observed in MCPs. Šídák’s multiple comparisons tests were performed between all concentrations independently, showing a *p* ≤ 0.0001 for the shift in 4 mg/mL samples and *p* ≤ 0.01 for the quenching at all concentrations.

Adding 0–20 mM of NaCh to a solution of 5 μM BSA in MQ water (Figure 7A) produced significant fluorescence quenching and a blue-shift in λ_max_ (Figure 7C,D,). By contrast, the same experiment with equal concentrations of PVA (Figure 7B) produced no significant effects in quenching or shift in λ_max_ (Figure 7C,D). Once our method was accurately developed, we tested the effect of different particle concentrations (2–4 mg/mL) on BSA under the same conditions. There is a strong effect on BSA caused by the CSPs (Figure 7E): BSA fluorescence was quenched by ∼30%, and the λ_max_ was shifted by 10–15 nm (Figure 7G,H). Conversely, MCPs showed minimal λ_max_ shifts and a slight quenching effect, possibly due to the increasing medium turbidity with growing NP concentrations (Figure 7G,H). Overall, these findings confirm the significant presence of NaCh on the surface of CSPs, sufficient to enhance interactions with BSA.

These results suggest that the greater protein adsorption of CSPs can be attributed to the presence of residual NaCh on their surface. Moreover, this observation indicates that after the homogenization phase, the interaction of NaCh with the PLGA matrix is strong enough to form stable core–shell structures that prevent its complete exchange with PVA. Adjusting parameters such as the evaporation time and stirring speed may allow for a tunable surfactant exchange process, enabling control over the surfactant ratio.

### 3.4 *In Vitro* Cell Uptake Experiments

**Figure 8:**
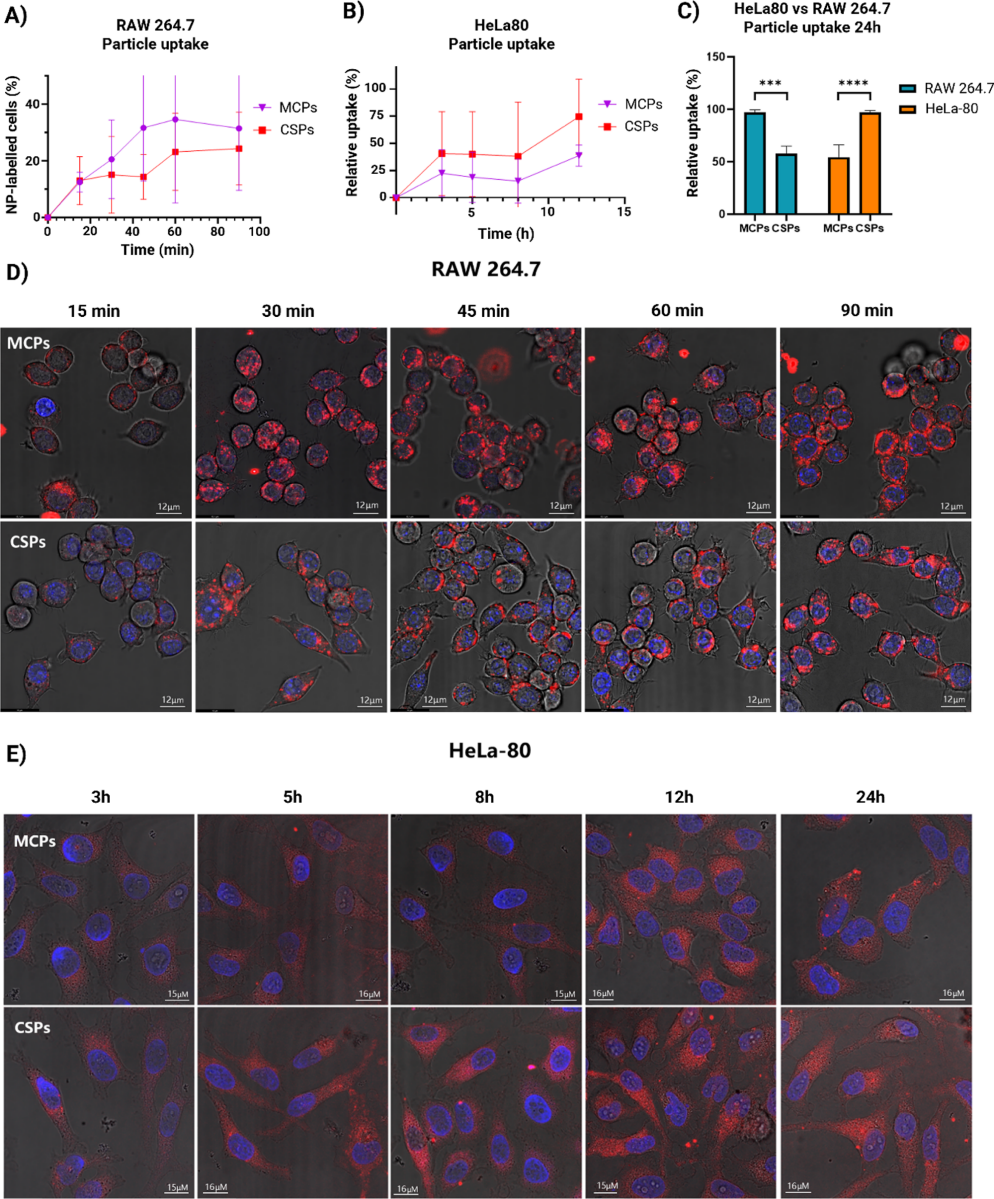
Uptake of MCPs and CSPs in RAW and HeLa cells at different time points. Particle uptake was quantified by flow cytometry over time during the early uptake process (15, 30, 45, 60, and 90 min for RAW cells and 3, 5, 8, and 12 h for HeLa cells) (A, B), and at an end point of 24 h (C). Particle uptake is represented with the NP-positive live cell population % in RAW cells and with the relative uptake % in HeLa cells and the 24 h plot, calculated using the median fluorescence of the live cell population, normalized to the highest value (as 100%). Independent *t* tests were conducted comparing CSPs vs MCPs uptake at 24 h for each cell type, showing significant differences in uptake between MCPs and CSPs in both cases (*p* ≤ 0.0001 in HeLa and *p* ≤ 0.001 in RAW; *n* ≥ 3). Confocal imaging (D, E) was performed to confirm the results observed by flow cytometry, showing NPs in red and the nuclei in blue (Hoechst), merged with the bright field view.

To better understand how particle structure and surface can affect particle–cell interactions and their potential impact *in vivo*, we studied the *in vitro* uptake profiles of both particle types using flow cytometry (Figure 8A–C) and confocal microscopy imaging (Figure 8D,E) with two distinct cell lines: phagocytic murine macrophages (RAW 264.7) and nonphagocytic human cancer cells (HeLa-80). These well-known and studied cell lines were chosen as representatives of immune and cancer cells with the aim of exploring how their phagocytic or nonphagocytic nature influences particle–cell interaction dynamics.

Particle uptake was investigated by measuring both the uptake rate over time and the total uptake at 24 h. The time points selected for each cell line were optimized according to their uptake behavior. Given the high internalization rate of RAW cells, measurements were taken at 15, 30, 45, 60, and 90 min, whereas for HeLa cells, which exhibited a slower uptake, time points of 3, 5, 8, and 12 h were selected. For these tests, particle batches with nearly equal fluorescence intensity were used.

*In vitro* particle uptake results are shown in Figure 8. For RAW macrophages, the initial uptake rates for MCPs and CSPs were similar, as confirmed by both flow cytometry (Figure 8A) and confocal microscopy (Figure 8D) analyses. However, a higher internalization of MCPs becomes apparent at longer incubation times (Figure 8A). The total uptake at 24 h (Figure 8C) was nearly double for MCPs, suggesting a clear preference for MCPs by RAW macrophages. Note that the uptake values presented in the 24 h graph are normalized relative to the highest fluorescence intensity observed for each cell type, designated as 100%. Therefore, the values do not represent the relative uptake between different cell lines but the relative uptake of different particle types for a given cell line.

In contrast, HeLa cells exhibited a preference for CSPs at all time points, as demonstrated by the uptake curves (Figure 8B) and total uptake at 24 h (Figure 8C) and also confirmed by the confocal images (Figure 8E). The uptake profile of HeLa cells exhibits a comparable pattern across both particle types, indicating that they likely utilize a similar mechanism for internalizing these particles. Therefore, we could assume that the differences in NP uptake by HeLa cells are driven by the efficiency of the internalization mechanism. In this case, the physical aspects, such as particle stiffness and protein corona, could play a major role in enhancing or hindering a specific uptake mechanism.

To further investigate the role of the protein corona in particle uptake, we performed uptake assays in serum-free media. This allowed us to distinguish the effects of particle stiffness and protein corona on the uptake efficiency. Since the lack of serum can induce cellular stress, potentially altering normal uptake processes, a WST-1 assay was conducted to assess cell viability and proliferation under serum-free conditions (Figure S5). RAW macrophages showed reduced viability after only 2 h in serum-free media; therefore, a 1 h incubation period was used for the uptake experiments. HeLa cells, however, maintained normal viability for up to 3 h in serum-free conditions, at which point uptake assays were analyzed.

**Figure 9:**
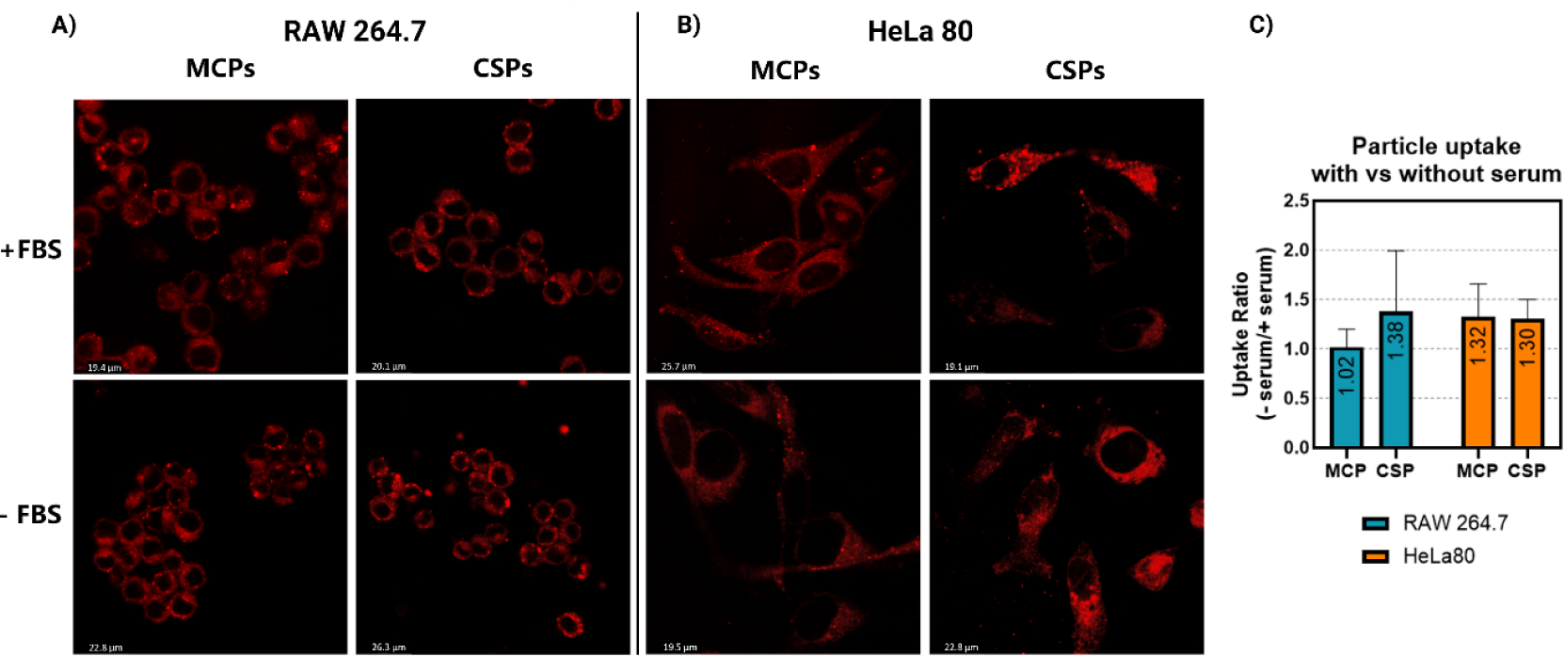
Particle uptake under normal vs serum-free conditions. Fluorescence microscopy images of RAW macrophages after 1 h (A) and HeLa cells after 3 h (B). NPs are shown in red. (C) Particle uptake, quantified by flow cytometry in serum-supplemented (10% FBS) and serum-free media for the selected time points. The graph depicts the uptake ratio (%) in serum-free conditions compared to serum-supplemented conditions. The mean ratio of both data sets was not significantly different from a theoretical mean of 1, as determined by one-sample *t* test statistical analysis.

Particle uptake under normal and serum-free conditions was monitored by confocal microscopy (Figure 9A,B) and quantified by flow cytometry analysis (*n* ≥ 3). The mean uptake ratio (calculated as uptake in the absence of serum divided by the uptake in the presence of serum) for each particle type and cell line is presented in Figure 9C. Although one-sample *t* tests showed no statistically significant differences in uptake compared to a theoretical mean of 1, we observed a general trend of enhanced uptake in serum-free conditions, suggesting that the absence of protein could facilitate particle–cell adhesion and internalization. Likewise, this effect could also be prompted by the stressing conditions in the absence of serum. HeLa cells showed an equal increase in uptake of both particle types in serum-free media, which indicates that the preference of HeLa cells for CSPs in serum-containing medium could be due to their stiffer structure, i.e., the effect of particle stiffness dominates over the particle surface characteristics for HeLa cells. Conversely, we could detect considerable differences in particle uptake by RAW cells. While MCPs showed an average of ∼1, revealing no influence of the serum in their uptake, the average uptake of CSPs by RAW macrophages increased by ∼38% in serum-free conditions. The observed trend is notable given the short incubation period (1 h). In alignment with our findings of lower protein adsorption on MCPs in serum-containing medium (Figure 5A,B), the lack of protein corona does not seem to influence their uptake efficiency by RAW cells in serum-free conditions. In contrast, for CSPs with significant protein adsorption (Figure 5A,B), the protein corona seems to be more influential on the efficiency of uptake by RAW cells as indicated by higher uptake in a serum-free medium (Figure 9C). Apparently, in the absence of a protein corona, CSPs are either more readily recognized or more efficiently internalized by the RAW macrophages.

Previous studies using similar particles on RAW macrophages have reported a preference for harder particles over softer ones,^19^ yet we observed a preference toward softer MCPs in serum-containing medium. Protein corona is known to hinder particle recognition by decreasing particle–cell membrane adhesion.^65^ Given the higher protein corona content of CSPs, it is likely that particle–cell interactions are compromised, negatively impacting their recognition and uptake. On the other hand, the low protein adsorption of MCPs barely affects their uptake, as confirmed by our serum-free experiments. This outcome aligns with other studies, where “naked” particles were more easily internalized by dendritic cells and macrophages compared to the same particles after protein adsorption.^30,66^

In contrast to RAW macrophages, our results indicate that HeLa cells are minimally affected by the protein corona thickness during uptake, with particle stiffness playing a more significant role. While most research focuses on smaller metallic nanoparticles in HeLa cells, limited literature exists on polymeric NPs of this size. Uptake in nonphagocytic cells, such as HeLa cells, is attributed to endocytic mechanisms, including macropinocytosis.^67^ This nonspecific form of endocytosis engulfs extracellular fluid and its contents into large vesicles (macropinosomes) and is often triggered by broader stimuli, such as serum.^68^ This process can explain the enhanced uptake of particles under serum-free conditions, the reduced influence of the protein corona, and the greater impact of particle stiffness on the uptake by HeLa cells. Studies demonstrate that the deformational energy required to wrap stiffer particles is overall smaller,^16,21^ supporting the preference for stiffer CSPs by HeLa cells.

Overall, the impact of different physicochemical properties of nanoparticles on cellular uptake is highly cell-type dependent. Our findings support the idea that the effect of particle stiffness on recognition by immune cells is highly dependent on the elastic range. While most studies demonstrate a significant impact with particles on the kPa range,^23,34,69^ particle stiffness within the MPa range has a minimal effect on macrophage uptake, as the overall rigidity of both MCPs and CSPs is high. Although nanoscale indentation experiments were implemented for mechanical characterization, the overall particle stiffness sensed by the cells is likely to be different. Nonetheless, we can conclude that the role of particle elasticity is overpowered by surface chemistry at such high stiffness ranges in macrophages (or immune cells), where the uptake is dependent on molecular recognition. Considering the multitude of factors influencing cell uptake and particle recognition, we note that these findings are specific to the studied particles in the RAW and HeLa cells. Further investigations of other cell types would be necessary to generalize these outcomes to similar cells.

Based on our observations, we hypothesize that the thinner protein corona on MCPs allows for easier recognition by immune cells, explaining their faster *in vivo* clearance compared to CSPs in previous studies.^11^ At the same time, the faster degradation and clearance of the MCPs from the retention organs could be attributed to the higher permeability and diffusion ability of the PFCE in the softer structure. Based on the evidence showed by Hartmann et al., softer particles are internalized and processed faster by the cell after their endocytosis,^22^ which in our case would also allow the PFCE to be cleared faster through circulation following the metabolization of the matrix components. From a clinical point of view, a faster degradation would reduce the risks associated with hepatic clearance and a sustained inflammatory response derived from the long-term accumulation of PFCs in the body.

## 4 Conclusions

In this study, we explored the structural and mechanical differences between two PFC-encapsulating polymeric NPs prepared using two different surfactants and their impact on particle–cell interactions. Aiming to analyze our particles from the core to the crust, we revealed the fundamental role of the surfactant in obtaining stable PFCE-loaded PLGA NPs. Our study establishes a direct link between the type of surfactant used in nanoparticle formulation and the resulting internal structure, stiffness, and protein corona, all of which significantly influence particle–cell interactions. While the impact of the surfactant on the protein corona might be anticipated, the formation of distinct internal nanoparticle structures and the associated differences in particle stiffness are particularly noteworthy.

These findings underscore the potential of engineering particle stiffness and architecture to achieve cell-specific delivery. By tailoring nanoparticles with different surfactant types, it becomes possible to fine-tune their ultrastructure, stiffness, and protein corona, thereby enhancing their uptake by specific cell types. In clinical applications, this approach could be harnessed to target specific immune cells, such as macrophages in inflammatory diseases or cancer cells for therapeutic purposes. Such customization paves the way for precision medicine, aligning nanoparticle design with patient-specific cellular and molecular profiles. Considering the immense challenge of developing stable perfluorocarbon nanoformulations, our insights offer a valuable foundation for the creation of versatile and clinically applicable PFC nanosystems.

## Supporting Information Available

Particle fluorescence quantification, NP-encapsulated PFCE quantification by NMR, application of Sneddon’s model, force curves obtained in liquid, ^1^H NMR spectra of CSPs and MCPs, and cell viability in serum-free conditions (PDF)

## Abbreviations

PLGA: poly(lactide-co-glycolide)
NPs: nanoparticles
PFCE: perfluoro-15-crown-5-ether
MCPs: multicore particles
CSPs: core–shell particles
PVA: poly(vinyl alcohol)
NaCh: sodium cholate
AFM: atomic force microscopy
BSA: bovine serum albumin
PFC: perfluorocarbon
MRI: magnetic resonance imaging
PPO: polypropylene oxide
SET: solvent evaporation technique
DLS: dynamic light scattering
QNM: quantitative nanomechanical mapping
NMR: nuclear magnetic resonance
DMSO: dimethylsulfoxide
DCM: dichloromethane
PFA: paraformaldehyde
TFA: trifluoroacetic acid
DMEM: Dulbecco’s modified Eagle Medium
SI: Supporting Information
PDI: polydispersity index
