# Supplementary material for: Perfluorocarbon-Loaded Poly(lactide-co-glycolide) Nanoparticles from Core to Crust: Multifaceted Impact of Surfactant on Particle Ultrastructure, Stiffness, and Cell Uptake
Source: ACS Appl Polym Mater. 2025 Mar 3;7(5):2864–78. doi: 10.1021/acsapm.4c03360 (PMC11915196; doi:10.1021/acsapm.4c03360)
Supplement: Supplementary file 1 — ap4c03360_si_001.pdf [file ap4c03360_si_001.pdf]

# Supporting Information

# Perfluorocarbon-Loaded Poly(lactide-*co*-glycolide) Nanoparticles from Core to Crust: Multifaceted Impact of Surfactant on Particle Ultrastructure, Stiffness, and Cell Uptake

*Naiara Larreina Vicente[1], Mangala Srinivas[1*], Oya Tagit[2*].*

1. Cell Biology and Immunology (CBI), Wageningen University, De Elst 1, 6708WD, Wageningen, Netherlands

2. Group of Biointerfaces, Institute for Chemistry and Bioanalytics, FHNW University of Applied Sciences and Arts Northwestern Switzerland, Hofackerstrasse 30, 4132 Muttenz, Switzerland

*Corresponding authors: Oya Tagit, oya.tagit@fhnw.ch; Mangala Srinivas, mangala.srinivas@wur.nl.

**Supporting Information File**: Figure S1 (Particle fluorescence quantification), Figure S2 (Fluorine content in MCPs and CSPs, measured by $^{19}$F NMR), Supplementary Text (Application of Sneddon's model), Figure S3 (Force curves obtained for MCPs and CSPs in liquid), Figure S4 ($^{1}$H NMR spectra of CSPs and MCPs), and Figure S5 (Cell viability in serum-free conditions).

**SUPPLEMENTARY FIGURE 1 (S1):** Particle fluorescence quantification.

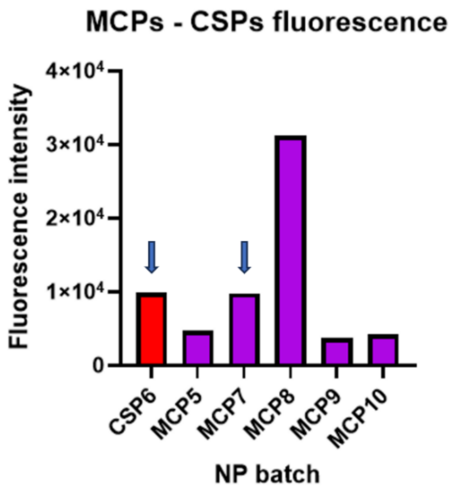


**Figure S1.** Fluorescence screening of different MCP batches against a selected batch of CSPs, labelled with AttoOxa12 at 1 mg/mL in PBS, measured with a plate reader (Ex: 626-30 nm, Em: 688-43 nm).

**SUPPLEMENTARY FIGURE 2 (S2)**: Fluorine content in MCPs and CSPs, measured by 19F NMR.

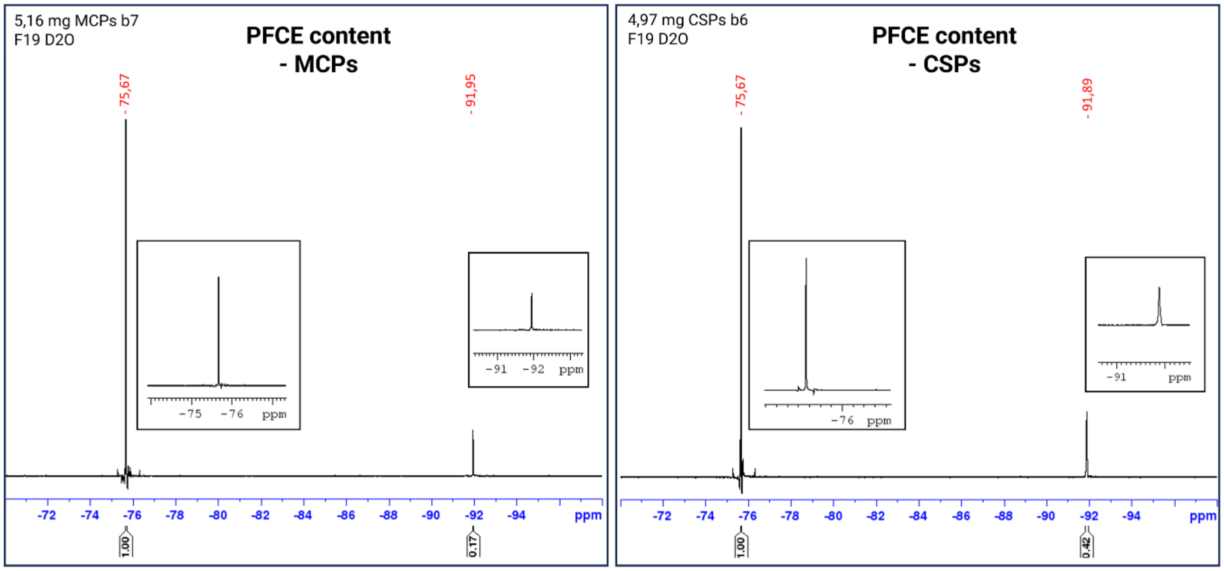


**Figure S2.** Representative $^{19}$F MRI spectra of two MCP and CSP batches. PFCE content is quantified using TFA as a reference. These spectra were obtained using a Bruker AV300 ultra shield NMR system, equipped with a PA BBO 300S1 BBF-H-D-05-Z probe.

**SUPPLEMENTARY TEXT:** Application of Sneddon's model.

This section details the experimental methods used to obtain the elastic modulus of the studied polymer NPs, as heterogeneous and complex samples.

The usual approach to extracting modulus information from force-distance curves involves the determination of the initial tip-sample contact point with subsequent analysis of tip penetration depth (indentation) through the sample. Because it can be challenging to precisely determine the exact contact point for certain samples (e.g. heterogeneous, soft, or in liquid- see Fig. S2), we used the linearized version of Sneddon's equation to fit the force curves.

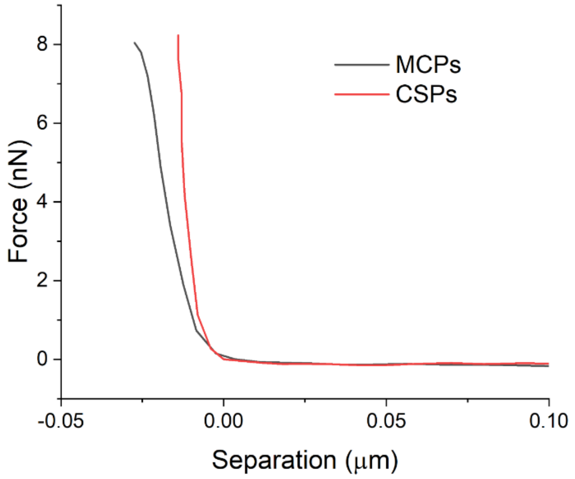


**Figure S3**. Approach curves obtained for MCPs (black) and CSPs (red) in liquid.

Sneddon's model describes the contact between a cone and an elastic half-space according to Eq. S1:

$$F = \frac{2}{\pi}\frac{E}{(1-v^2)}\tan(\alpha)\delta^2 \qquad \text{(Eq. S1)}$$

where;

F = force

E = Young's modulus

ν = Poisson's ratio

α = half-angle of the indenter

δ = indentation

We set the Poisson's ratio as 0.5, and the half angle of the indenter as 18° for our analyses. Eq. S1 may be linearized by taking the 1/2 power of both sides (Eq. S2), which is no longer forced through the origin (0,0); therefore, determining the contact point is no longer needed for the analysis. Instead, Young's modulus is calculated from the slope of the curves via selecting the fit region.

$$(F)^{1/2} = \left(\frac{2}{\pi}\frac{E}{(1-\nu^2)}\tan(\alpha)\right)^{1/2}\delta \qquad \text{(Eq. S2)}$$

We fit the model on the approach curves by setting the lower and upper fit boundaries to approximately 10 % and 70 % of the maximum exerted force, respectively. Using approach curves (instead of retraction curves) ensures that nanomechanical properties are probed without the influence of any prior deformation. In addition, the analysis is less affected by the potential influence of adhesion forces. Overall, the utilization of the linearized Sneddon model to fit the approach curves could reflect the local stiffness and elastic modulus of our samples with complex and heterogeneous structures. Also, to account for heterogeneity, only the approach curves obtained on the top-middle part of the nanoparticles were analyzed and fit values satisfying $R^2 > 0.99$ were averaged to determine the apparent Young's modulus of elasticity of MCPs and CSPs in air and liquid.

**SUPPLEMENTARY FIGURE 4 (S4):** $^{1}$H NMR spectra of CSPs and MCPs.

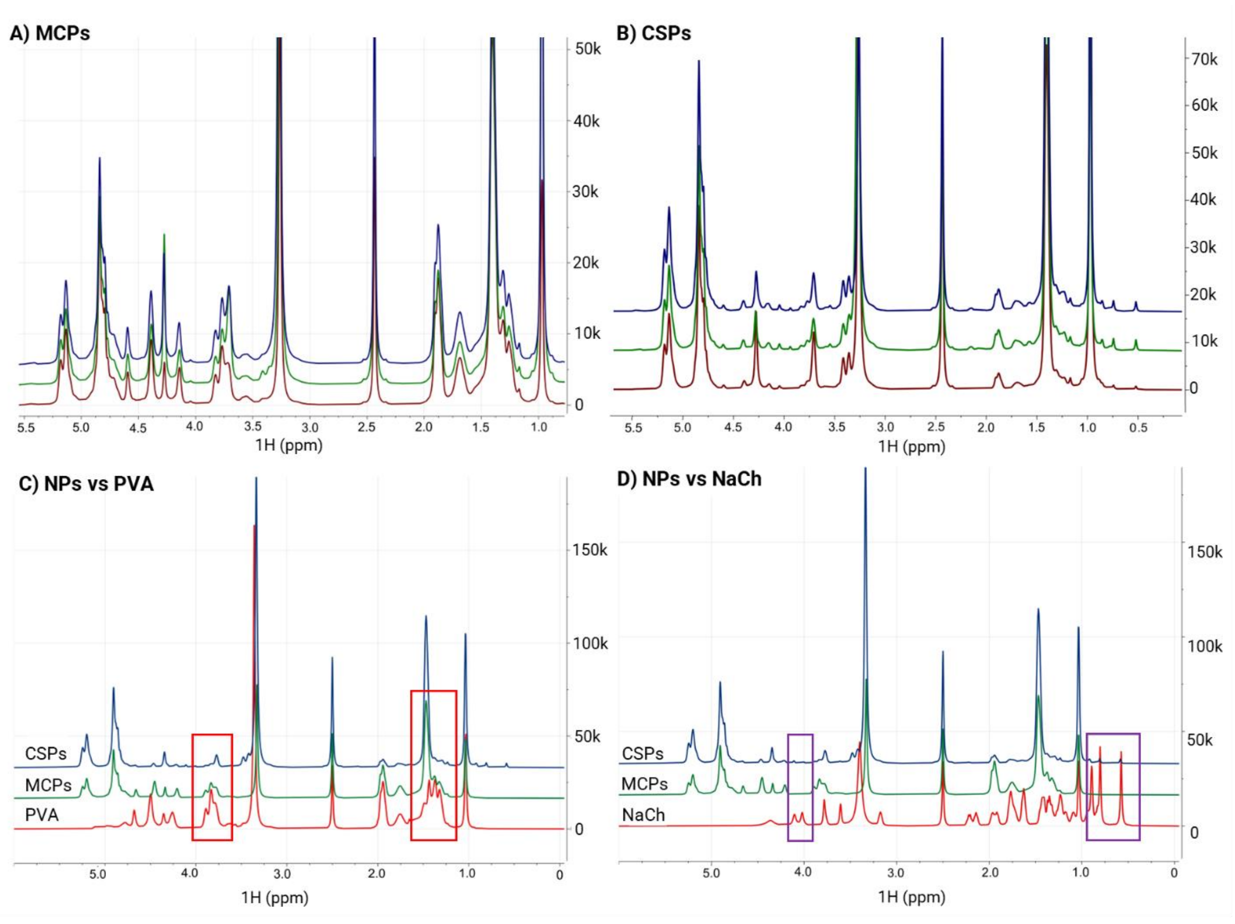


**Figure S4**. $^{1}$H NMR analysis of MCPs, CSPs, and surfactants (PVA and NaCh). $^{1}$H NMR spectra of three different batches of MCPs (A) and CSPs (B) dissolved in DMSO-$d_6$ are aligned showing equal spectrums and composition consistency between batches. Plots C and D display aligned spectra of MCPs, CSPs and the surfactants PVA and NaCh, respectively. In each plot, overlapping peaks are highlighted, revealing higher PVA content in MCPs (C) and the presence of NaCh in CSPs, as well as the absence of these peaks in MCPs (D).

**SUPPLEMENTARY FIGURE 5 (S5):** Cell viability in serum-free conditions.

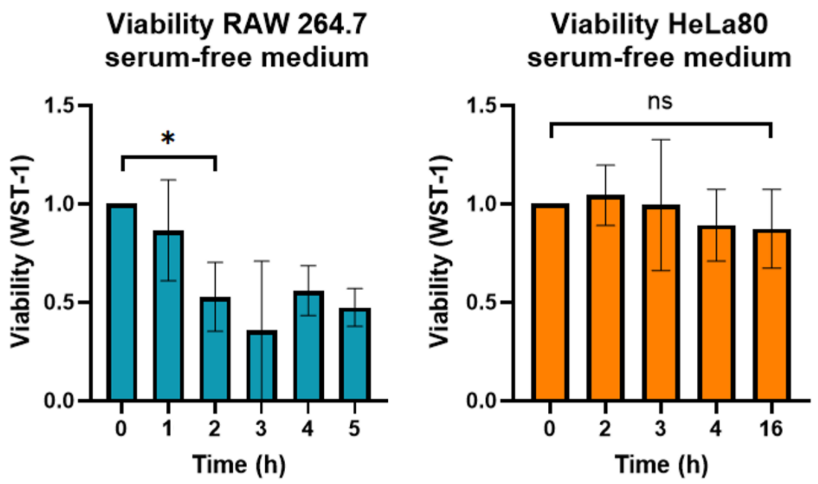


**Figure S5.** Viability of RAW 264.7 (blue) and HeLa-80 (orange) cell lines in serum-free medium, assessed using a colourimetric assay (WST-1). Viability was significantly reduced in RAW macrophages after only 2h, while HeLa cells showed no significant drop in viability even after 16h. The absorbance values were normalised to the control sample, and an ordinary one-way ANOVA with multiple comparisons was conducted for statistical analysis. 1 h and 3 h incubation times were chosen for the serum-free uptake experiments, respectively.
